# B-lymphoblastic leukemia/lymphoma with *MYC* and *BCL2* gene rearrangements shows evidence for clonal evolution and mitotic recombination

**DOI:** 10.1007/s12308-023-00541-y

**Published:** 2023-04-20

**Authors:** Steven A. Schichman, Andrea L. Penton, Sai Nikhila Ghanta, Manojna Konda, Peter R. Papenhausen

**Affiliations:** 1 Central Arkansas Veterans Healthcare System, Pathology and Laboratory Medicine Service, and Department of Pathology, University of Arkansas for Medical Sciences, Little Rock, AR USA; 2 Cytogenetics Department, Laboratory Corporation of America, Research Triangle Park, NC USA; 3 Department of Internal Medicine, University of Arkansas for Medical Sciences, Little Rock, AR USA; 4 Department of Internal Medicine, Division of Hematology and Oncology, University of Arkansas for Medical Sciences, Little Rock, AR USA

**Keywords:** B-lymphoblastic leukemia/lymphoma, Clonal evolution, Mitotic recombination

## Abstract

**Background:**

B-lymphoblastic leukemia/lymphomas (B-ALL/LBL) are uncommon neoplasms that may be associated with a variety of cytogenetic and molecular changes. The mechanisms by which these changes arise have not been fully described.

**Aims/Purpose:**

This report describes an unusual case of B-ALL/LBL with complex clonal evolution that includes BCL2 and MYC gene rearrangements.

**Methods:**

Immunophenotyping was performed by immunohistochemistry and flow cytometry. Traditional G-band karyotyping was accompanied by fluorescence in-situ hybridization (FISH) using break-apart and dual fusion probes. Single nucleotide polymorphisms were assessed using a high-density DNA microarray.

**Results:**

The karyotype of the blasts showed reciprocal translocation of chromosomes 4 and 18, reciprocal translocation of chromosomes 8 and 14 with two copies of the oncogenic translocation derivative(14)t(8;14), and no normal chromosome 14. FISH studies showed complex IGH-BCL2 and IGH-MYC fusion signals.

**Conclusions:**

A clonal evolution model involving multiple chromosomal translocations and mitotic recombination is postulated to account for the karyotype, FISH, and microarray results but leaves unresolved the exact order of the evolutionary changes.

## Introduction

Aggressive hematologic malignancies sometimes harbor multiple structural and/or numerical chromosomal abnormalities detected by cytogenetics, FISH, or microarray techniques. These abnormalities are thought to arise from clonal evolution in which accumulation of genetic changes confers growth advantage to subclones of the original tumor leading to tumor progression [1]. Hematolymphoid neoplasms often show evidence of clonal evolution in their karyotypes and/or gene mutational profiles either at diagnosis or relapse after chemotherapy [2–4]. This report describes an unusual case of B-ALL/LBL with complex *MYC* and *BCL2* gene rearrangements. A step-wise process for clonal evolution of the disease is proposed based on cytogenetic, FISH, and microarray studies.

## Clinical history

An 83-year-old man presented with complaints of generalized weakness, dizziness, and 30-pound weight loss over 2 months. His medical history was negative for leukemia or lymphoma. Computerized axial tomography of the chest, abdomen, and pelvis showed neither lymphadenopathy nor hepatosplenomegaly. Laboratory analysis showed hemoglobin 13.5 g/dl, platelet count 36,000/µl, and white blood cell count 11,900/µl with 56% neutrophils, 15% bands, 5% myelocytes, 1% monocytes, 10% lymphocytes, and 13% blasts. After diagnosis, the patient declined treatment and transitioned to hospice care.

## Materials and methods

### Immunohistochemistry

Immunohistochemical staining was performed on a Ventana Benchmark Ultra system using validated automated operating protocols. Ventana monoclonal antibodies used were CD20 (L26), PAX-5 (SP34), CD3 (2GV6), BCL-2 (124), MYC (Y69), and Ki-67 (SP6). Cell Marque monoclonal antibodies used were CD10 (56C6) and BCL6 (G191E/A8).

### Flow cytometry

Flow cytometry of the bone marrow aspirate was performed by Accupath Diagnostic Laboratories (Brentwood, TN, USA) using 8-color flow analysis performed on a Becton–Dickinson (BD) FACSLyric™ flow cytometer.

### Karyotype and FISH

Chromosome analysis and FISH were performed on the same bone marrow aspirate by a reference laboratory (Integrated Oncology, RTP, NC, USA). FISH was performed using break-apart probes for *MYC*, *BCL2*, *BCL6*, *PDGFRA* and dual fusion probes targeting *IGH*-*MYC* and *IGH-BCL2* (Kreatech, Leica Biosystems). Breakapart FISH probes were used in the initial screening for gene rearrangements. Dual fusion FISH probes were used to characterize specific gene rearrangements.

### DNA microarray

The Cytoscan HD chip and GeneChip instrument system (Affymetrix, Thermo Fisher Scientific) was used for whole genome single nucleotide polymorphism (SNP) microarray analysis. Propriety software (CHAS) from Applied Biosystems was utilized for chromosome analysis of microarray data to determine SNP genotypes based on the GRCh37/hg19 assembly.

## Results

### Bone marrow biopsy and immunophenotyping

**Fig. 1:**
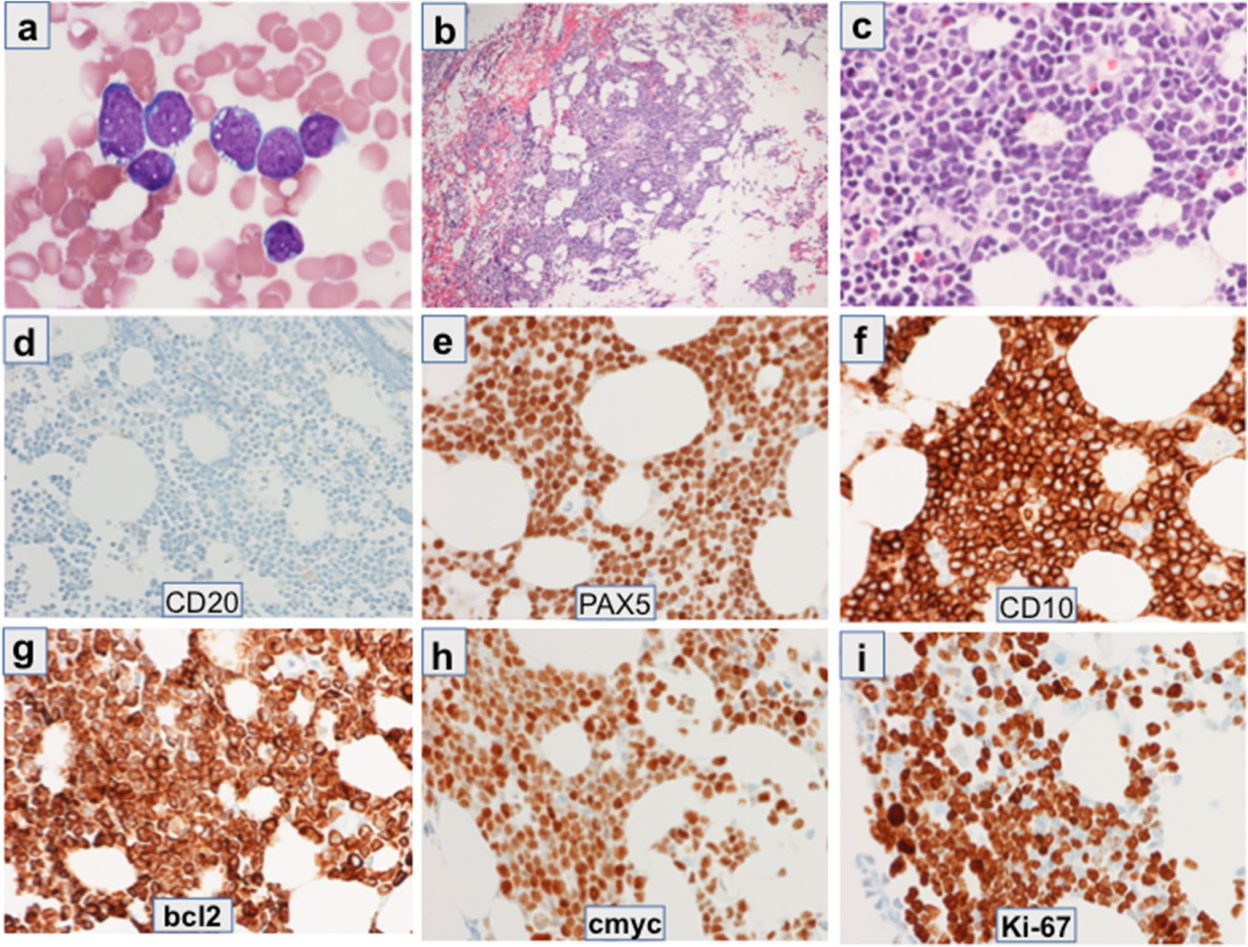
Bone marrow aspirate and bone marrow core biopsy with immunohistochemical stains: (**a**) Bone marrow aspirate, Wright-Geimsa stain, 1000 × magnification, (**b**) Core biopsy, hematoxylin and eosin stain, 200 × magnification, (**c**) Core biopsy, hematoxylin and eosin stain, 600 × magnification, (**d**–**i**) Immunohistochemical stains of the core biopsy with markers indicated within each panel

Bone marrow aspirate smears showed approximately 70% blasts. Blasts were variable in size with immature chromatin, indistinct nucleoli, deep blue cytoplasm and prominent cytoplasmic vacuoles (Fig. 1a). A few mitotic figures were noted. A hypercellular bone marrow core biopsy showed leukemic infiltration by blasts (Fig. 1b and c) corresponding to those in the aspirate smear. Some residual hematopoiesis was present. Selected immunohistochemical stains are shown in Fig. 1d–i. Blasts were positive by immunohistochemistry for PAX5, CD10, BCL2, MYC, and BCL6 (subset) with approximately 90% of the blasts positive for Ki-67. Blasts were negative for CD20, CD3, and EBER-ISH. Flow cytometry of the peripheral blood and bone marrow showed a population of B cells that expressed CD45(dim), HLA-DR, CD19, CD10, CD22, cCD22, cCD79a(+ / −), and CD38. The B cells were negative for CD20, CD34, TdT, sIg, cIg, cCD3, MPO, CD13, CD33, CD117, CD11b, CD11c, CD14, CD64, CD15, CD56, CD2, CD3, CD5, and CD7.

### Karyotype and FISH

**Fig. 2:**
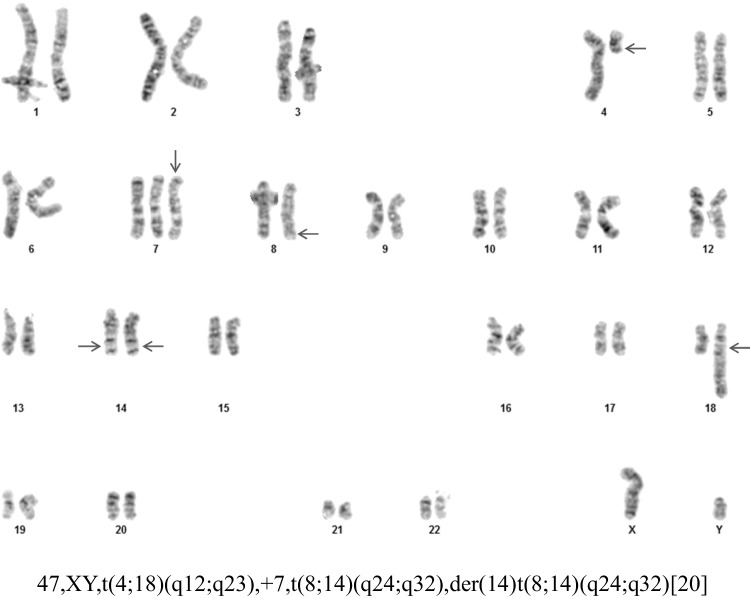
G-band karyotype of the bone marrow aspirate. Arrows indicate abnormal chromosomes

**Fig. 3:**
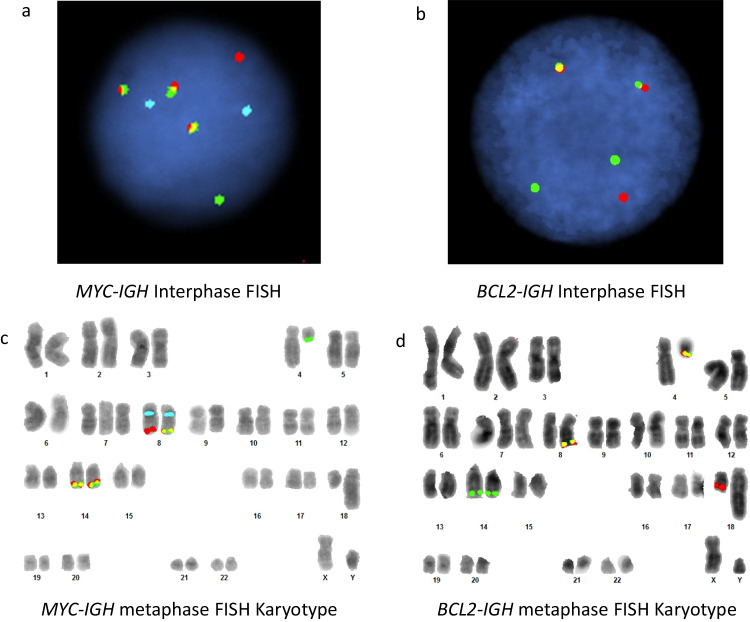
FISH of the bone marrow aspirate: (**a**) Interphase FISH with dual fusion *MYC* (red), *IGH* (green), and centromere 8 (blue) probes, (**b**) Interphase FISH with dual fusion *BCL2* (red) and *IGH* (green) probes, (**c**) Metaphase FISH karyotype with dual fusion *MYC* (red), *IGH* (green), and centromere 8 (blue) probes, (**d**) Metaphase FISH karyotype with dual fusion *BCL2* (red) and *IGH* (green) probes

Cytogenetic analysis of GTG banded metaphases from B-mitogen stimulated cultures of the bone marrow aspirate (Fig. 2) showed 47,XY,t(4;18)(q12;q23), + 7,t(8;14)(q24;q32),der(14)t(8;14)(q24;q32) [20]. The karyotype of the abnormal lymphoid clone was characterized by a reciprocal translocation involving chromosomes 4 and 18 and a reciprocal translocation involving chromosomes 8 and 14. In addition, all cells contained an extra translocation derivative 14 of the t(8;14) in place of the normal chromosome 14 as well as an extra copy of chromosome 7. Interphase FISH of bone marrow aspirate showed that 92% of the cells were positive for three *MYC-IGH* fusion signals (Fig. 3a). Interphase FISH also showed that 89% of the cells were positive for two *BCL2-IGH* fusion signals (Fig. 3b). FISH was negative for *BCL6* and *PDGFRA* rearrangement. A more detailed analysis was performed by constructing *MYC-IGH* and *BCL2-IGH* metaphase FISH karyotypes (Fig. 3c and d). In Fig. 3c, the *MYC-IGH* fusions (yellow) are present on two identical copies of the derivative 14 and the translocation derivative 8. In addition, a *MYC* signal (red) is present on the normal 8 and an *IGH* signal (green) is seen on the translocation derivative 4. In Fig. 3d, the *BCL2-IGH* fusions (yellow) are present on the 4 and 8 translocation derivatives. A *BCL2* signal (red) is present on the normal 18 and *IGH* signals (green) are present on the derivative 14 chromosomes.

### SNP microarray

Whole genome SNP microarray analysis showed allele heterozygosity of chromosome 14 from the centromere to the immunoglobin heavy chain locus near the long arm terminus. In addition, the array demonstrated a gain of all of chromosome 7 (detected also by karyotype), a gain of distal 8q from the *MYC* gene at q24.21 to the telomere, and three microdeletions in chromosome 9. The latter included an approximately 0.5 Mb single copy loss at 9p13.2 spanning the *PAX5* gene and bi-allelic deletions of different sizes at 9p21.3 spanning the *CDKN2A* tumor suppressor. The SNP microarray also showed an extended contiguous region of allele homozygosity in the p terminus of chromosome 6 consistent with acquired copy-neutral loss of heterozygosity (CN-LOH) (data not shown).

## Discussion

Precursor B-cell neoplasms with *IGH-MYC* rearrangement have been well documented [5, 6]. The leukemic B-cell neoplasm described in this case report, however, has unique features that make it difficult to classify. The clinical presentation, precursor B-cell immunophenotype (CD45(dim), CD19, PAX5, CD10, CD22, cCD22, and cCD79a), negative CD20, and karyotype/FISH results are consistent with a diagnosis of either “B-ALL with *MYC* rearrangement” in the 2022 ICC classification [7] or “B-ALL/LBL with other defined genetic abnormalities” in the 2022 WHO classification [8]. The absence of immunoglobulin expression and deletion of *CDKN2A*, although not specific for B-ALL/LBL, lend support to the diagnosis. However, the negative TdT and expression of BCL6 in a subset of malignant cells raise the possibility of a high grade B-cell lymphoma with *MYC* and *BCL2* rearrangements [9] or a lymphoblastic transformation of follicular lymphoma [10], although there is no clinical evidence to support these diagnoses.

**Fig. 4:**
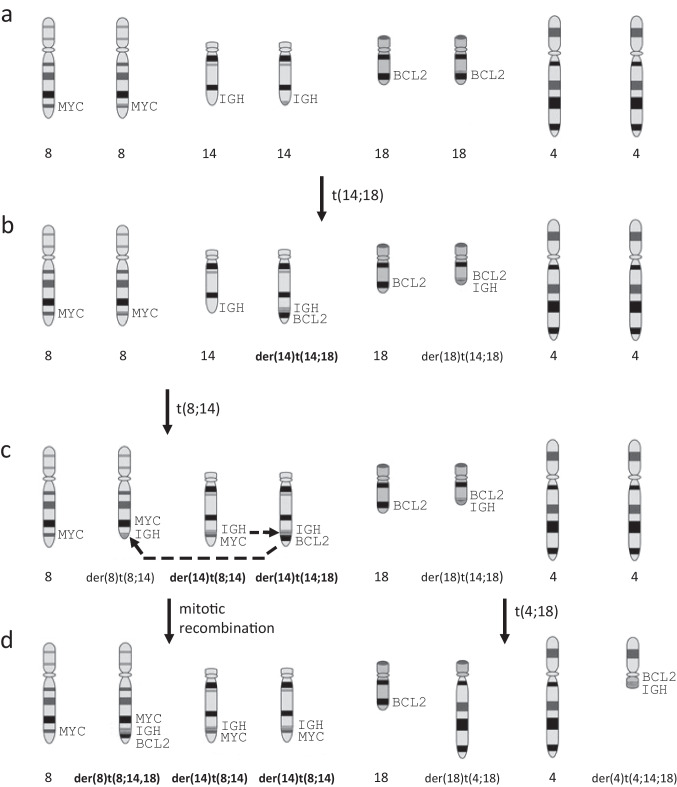
Stepwise model for clonal evolution of the patient’s B-ALL involves reciprocal translocations and mitotic recombination. Oncogenic translocation derivatives are indicated in bold type: (**a**) Normal chromosome pairs 8, 14, 18 and 4 with chromosomal locations of *MYC* (8q24), *IGH* (14q32), and *BCL2* (18q21) indicated, (**b**) Reciprocal translocation of chromosomes 14 and 18, (**c**) Reciprocal translocation of chromosomes 8 and 14, (**d**) Mitotic recombination involving the two translocation derivative 14 chromosomes with displacement of the *BCL2-IGH* fusion from the der(14)t(14;18) to the translocation derivative 8 chromosome as indicated by dashed arrows in 4c; reciprocal translocation of chromosome 4 and der(18)t(14;18)

The complex karyotype and FISH results shown in Figs. 2 and 3 are strongly suggestive of multistep leukemogenesis driven by clonal evolution. Postulated intermediate steps in the clonal evolution process are illustrated in Fig. 4. The first step is proposed to be a t(14;18)(q32;q21) reciprocal translocation that results in the juxtaposition of the *BCL2* and *IGH* loci with the oncogenic driver on the der(14)t(14;18) (Fig. 4b). The second step is postulated to be t(8;14)(q24;q32) reciprocal translocation which causes juxtaposition of the *MYC* and *IGH* loci with the oncogenic driver on the der(14)t(8;14) (Fig. 4c). The next steps are proposed to be der(14) mitotic recombination (MR) and t(4;18)(q12;q23) reciprocal translocation. The MR duplicates the *MYC*-*IGH* fusion from the der(14)t(8;14) to its homologous chromosome (Fig. 4d). It is postulated from the targeted FISH studies that this MR displaced the oncogenic *BCL2*-*IGH* fusion from the der(14)t(14;18) to the der(8)t(8;14) distal to the non-oncogenic *MYC*-*IGH* fusion (see dashed arrows in Fig. 4c) to create the G-band cryptic der(8)t(8;14;18). The t(4;18)(q12;q23) presumptively involved an original der(18)t(14;18) and a normal copy of chromosome 4 to create a cryptic der(4)t(4;14;18) and der(18)t(4;18) (Fig. 4d). Because it is not possible to determine the order of the t(4;18) and der(14) MR events, these two steps in the evolution of the neoplastic clone are illustrated as occurring simultaneously in Fig. 4d.

Although the proposed model of clonal evolution in Fig. 4 accounts for the karyotype and FISH data, it is not possible to determine with certainty the sequence of events because intermediate clones in the evolution of the patient’s leukemia were not extant at clinical presentation of the disease and no subclones were detected by cytogenetics. It is logical, however, to start the clonal evolution sequence with a low-grade t(14;18) translocation followed by a high-grade t(8;14) translocation and duplication of the der(14)t(8;14) chromosome. In this context, it is unclear how the t(4;18)(q12;q23) translocation may lead to a selective growth advantage of the leukemic clone. One mechanism may be activation of an oncogene at the translocation breakpoint. To investigate this possibility, *PDGFRA* at 4q12 was tested and ruled out by FISH. However, activation of an unknown oncogene at 4q12 or 18q23 may be postulated. Alternatively, the t(4;18) translocation may represent a sporadic event that does not confer a growth advantage to the clone.

The B-ALL/LBL case in this report is unique because the *BCL2* rearrangement was detected by FISH without the t(14;18) appearing in the karyotype, secondary to separate rearrangements of both translocation derivatives. The novel retention of the oncogenic *BCL2*-*IGH* fusion, apparently moved from the der(14)t(14;18) to the der(8)t(8;14;18), is also a unique feature of this case because nearly all MR leads to loss of the displaced segment. The proposed model provides insight into possible mechanisms of clonal evolution in leukemia and other hematologic malignancies.
